# Identification of a Prognostic Signature Associated With the Homeobox Gene Family for Bladder Cancer

**DOI:** 10.3389/fmolb.2021.688298

**Published:** 2021-07-21

**Authors:** Bingqi Dong, Jiaming Liang, Ding Li, Wenping Song, Jinbo Song, Mingkai Zhu, Shiming Zhao, Yongkang Ma, Tiejun Yang

**Affiliations:** ^1^ Department of Urology, Affiliated Cancer Hospital of Zhengzhou University, Henan Cancer Hospital, Zhengzhou, China; ^2^ State Key Laboratory of Respiratory Disease, National Clinical Research Center for Respiratory Disease, The First Affiliated Hospital of Guangzhou Medical University, Guangzhou, China; ^3^ Department of Pharmacy, Affiliated Cancer Hospital of Zhengzhou University, Henan Cancer Hospital, Zhengzhou, China

**Keywords:** bladder cancer, homeobox gene family, prognostic signature, immunotherapy, biomarkers

## Abstract

**Background:** Bladder cancer (BLCA) is a common malignant tumor of the genitourinary system, and there is a lack of specific, reliable, and non-invasive tumor biomarker tests for diagnosis and prognosis evaluation. Homeobox genes play a vital role in BLCA tumorigenesis and development, but few studies have focused on the prognostic value of homeobox genes in BLCA. In this study, we aim to develop a prognostic signature associated with the homeobox gene family for BLCA.

**Methods:** The RNA sequencing data, clinical data, and probe annotation files of BLCA patients were downloaded from the Gene Expression Omnibus database and the University of California, Santa Cruz (UCSC), Xena Browser. First, differentially expressed homeobox gene screening between tumor and normal samples was performed using the “limma” and robust rank aggregation (RRA) methods. The mutation data were obtained with the “TCGAmutation” package and visualized with the “maftools” package. Kaplan–Meier curves were plotted with the “survminer” package. Then, a signature was constructed by logistic regression analysis. Gene Ontology (GO) and Kyoto Encyclopedia of Genes and Genomes (KEGG) analyses were performed using “clusterProfiler.” Furthermore, the infiltration level of each immune cell type was estimated using the single-sample gene set enrichment analysis (ssGSEA) algorithm. Finally, the performance of the signature was evaluated by receiver-operating characteristic (ROC) curve and calibration curve analyses.

**Results:** Six genes were selected to construct this prognostic model: TSHZ3, ZFHX4, ZEB2, MEIS1, ISL1, and HOXC4. We divided the BLCA cohort into high- and low-risk groups based on the median risk score calculated with the novel signature. The overall survival (OS) rate of the high-risk group was significantly lower than that of the low-risk group. The infiltration levels of almost all immune cells were significantly higher in the high-risk group than in the low-risk group. The average risk score for the group that responded to immunotherapy was significantly lower than that of the group that did not.

**Conclusion:** We constructed a risk prediction signature with six homeobox genes, which showed good accuracy and consistency in predicting the patient’s prognosis and response to immunotherapy. Therefore, this signature can be a potential biomarker and treatment target for BLCA patients.

## Introduction

Bladder cancer (BLCA) is a common urological tumor, and its morbidity and mortality rates are increasing year by year (Siegel et al., 2019). High recurrence and early metastasis lead to the poor prognosis of BLCA. The detection of exfoliated tumor cells in urine or bladder lavage samples has a high sensitivity (84%) for the diagnosis of high-grade BLCA but is less sensitive for low-grade BLCA (Babjuk et al., 2019). Cystoscopy, the main method for the diagnosis of BLCA, is invasive, time-consuming, and tedious. Currently, specific, reliable, and non-invasive tumor biomarker tests for the diagnosis and prognosis evaluation of BLCA are desperately needed.

The homeobox gene family is a group with a homologous segment of approximately 180 bp in length that encodes a homologous domain of 60 amino acids and is an important transcriptional regulator that plays a vital role in tumor formation and development, regulating cell proliferation, migration, and apoptosis (Laughon and Scott, 1984; Srebrow et al., 1998; Yang et al., 2015). Current studies have shown that the homeobox gene family is aberrantly expressed in different tumors, such as bladder, bile duct, endometrial, and breast cancers (Rao et al., 2002). In BLCA, ISL1 and LHX5 play important roles in multiple stages of bladder tumorigenesis (Akhir et al., 2020); ZHX3 promotes migration and invasion *in vitro* and *in vivo* (Deng et al., 2021). Therefore, the homeobox gene family plays an important role in the development and progression of BLCA. Although progress has been made in the study of individual family members, the role and prognostic value of the homeobox gene family in BLCA remain unclear.

**FIGURE 1:**
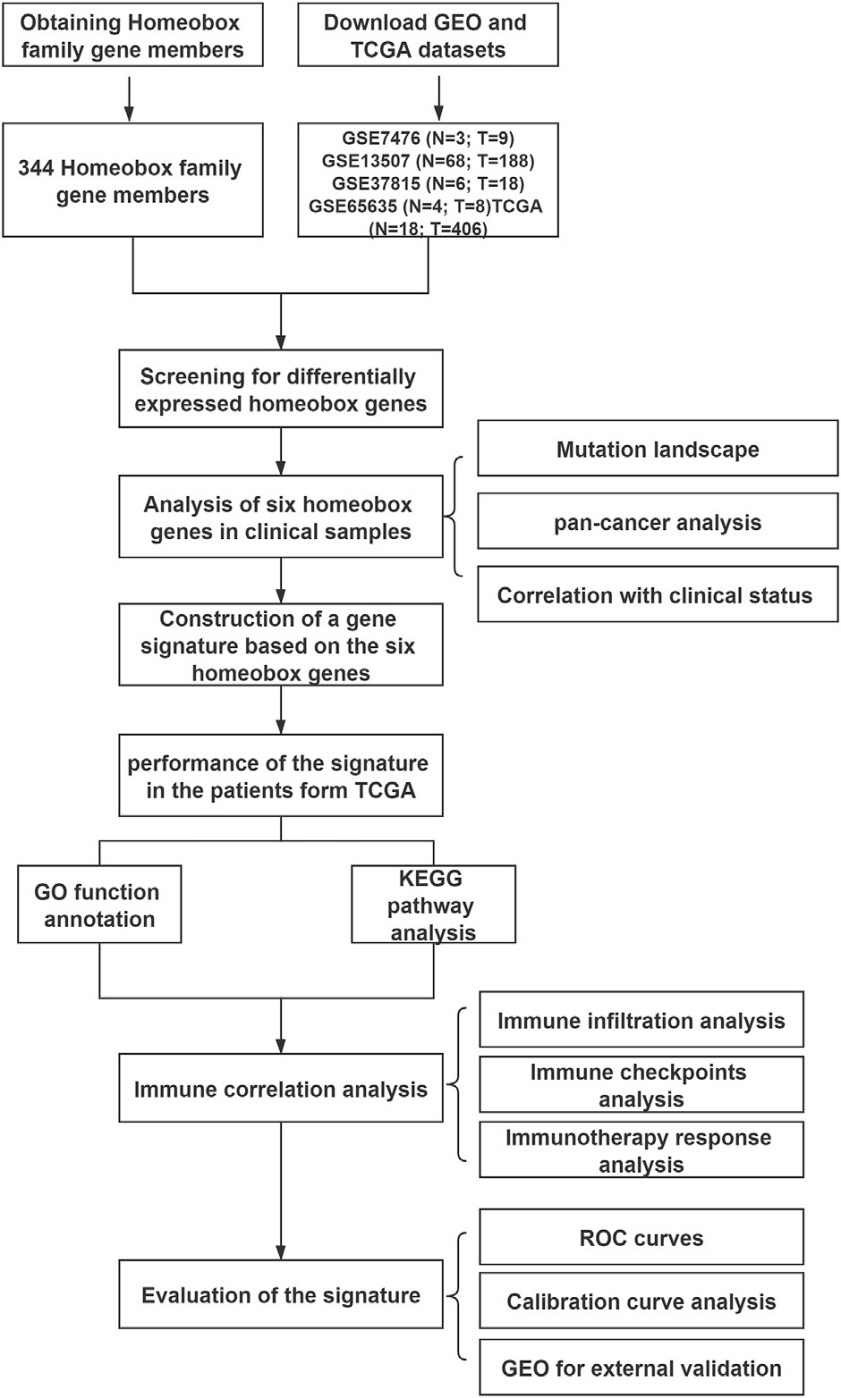
Flow chart showing the design of the study, with GSE7476 (*N* = 3; T = 9), GSE13507 (*N* = 68; *T* = 188), GSE37815 (*N* = 6; *T* = 18), GSE65635 (*N* = 4; T = 8), and TCGA (*N* = 18; *T* = 406) datasets.

In this study, we analyzed the mRNA expression of a large number of BLCA samples in public databases [The Cancer Genome Atlas (TCGA) and Gene Expression Omnibus (GEO)]. We constructed a prognostic signature for BLCA based on six homeobox genes with significant differential expression between BLCA tissues and normal tissues. This signature can predict a patient's prognosis and response to immunotherapy and thus has good clinical application value. The design flow chart for the entire analysis process of this study is shown in Figure 1.

## Materials and Methods

### Data Collection

The RNA sequencing (RNA-Seq) data, clinical data, and probe annotation files of BLCA patients (providing 18 normal tissues and 406 tumor tissues) in TCGA were downloaded from the University of California, Santa Cruz (UCSC), Xena Browser (https://xenabrowser.net/). BLCA datasets GSE7476 (3 normal tissues and 9 tumor tissues), GSE13507 (69 normal tissues and 188 tumor tissues), GSE37815 (6 normal tissues and 18 tumor tissues), GSE65635 (4 normal tissues and 8 normal tissues), and GSE19423 (48 tumor tissues) were downloaded from the Gene Expression Omnibus (GEO) database (https://www.ncbi.nlm.nih.gov/geo/) using the R package “GEOquery” (Davis and Meltzer, 2007). All 344 homeobox gene family members were extracted from the Hugo Gene Nomenclature Committee (HGNC). The probe IDs in each BLCA dataset were transformed into gene symbols according to the annotation files.

### Identification and Integration of Differentially Expressed Genes

The R package “limma” was used to identify DEGs between normal and tumor tissues in each BLCA cohort with cutoff criteria of adjusted *p* value <0.05 and |log fold change (FC)| > 0.5 (Ritchie et al., 2015). DEGs acquired from the five BLCA cohorts were sorted by the log fold change (logFC) value, and then the five gene lists were integrated using the RobustRankAggreg (RRA) R package (Kolde et al., 2012). The RRA method is based on the assumption that if the gene rank is high in all datasets, the probability that the gene is differentially expressed is higher and the related *p* value is lower.

### Mutation Landscape Analysis

TCGA BLCA mutation data containing 411 tumor samples were acquired from the R package “TCGAmutations.” The mutation landscape for the six signature genes in BLCA was visualized using the R package “Maftools” (Mayakonda et al., 2018).

### Construction and Evaluation of the Prognosis Model

We randomly divided the TCGA BLCA cohort (*n* = 406) in a 7:3 ratio into a training dataset (*n* = 285) and a testing dataset (*n* = 121). Logistic regression analysis was used to integrate the prognostic value of the six homeobox family genes into a six-gene signature model for BLCA. The formula for calculating the risk score for each sample is as follows:

Risk score= 0.062×TSHZ3+0.122×ZFHX4−0.031×ZEB2−0.206×MEIS1+0.012×ISL1−0.061×HOXC4.

We calculated the risk score using the expression profiles of each sample based on the formula of the signature model. Then, we divided the BLCA cohort into high- and low-risk groups based on the median risk score. The R package “survival ROC” was used to establish the receiver-operating characteristic (ROC) curves for predicting one-, three-, and five-year overall survival (OS) for the two risk groups. Furthermore, we used the R package “rms” to construct calibration curves and evaluate the precision of the one-, three-, and five-year OS predictions for the BLCA cohort.

### Estimation of Immune Cell Infiltration

We identified a group of 782 genes that represent 28 immune cell types involved in innate and adaptive immunity to estimate the infiltration level of different immune cell types in the tumor microenvironment (Charoentong et al., 2017). Subsequently, the single-sample gene set enrichment analysis (ssGSEA) algorithm with the R package “GSVA” was used to evaluate the infiltration level of each immune cell type based on the expression profiles of each sample in BLCA and the immune cell gene marker (Hänzelmann et al., 2013).

### Functional Enrichment Analysis

The Gene Ontology (GO) and Kyoto Encyclopedia of Genes and Genomes (KEGG) databases include collections of gene sets associated with the function of cells and organisms. Functional enrichment analysis of a set of genes that are dysregulated under certain conditions revealed which GO terms or KEGG pathways are overrepresented for that gene set. The TCGA BLCA cohort was divided into high-risk and low-risk groups according to the median risk score. Then, the R package “limma” was used to identify DEGs between the two risk groups. GO and KEGG analyses of the DEGs between the two risk groups were performed using the R package “clusterProfiler” (Yu et al., 2012). A cutoff value of adjusted *p* value < 0.05 was used to determine the significant pathways.

### Prediction of the Immunotherapy Response

The response of each sample to PD-1/PD-L1 and CTLA4 inhibitors was evaluated according to the gene expression profiles of the BLCA cohort with the Tumor Immune Dysfunction and Exclusion (TIDE) algorithm (http://tide.dfci.harvard.edu) (Jiang et al., 2018).

### Survival Analysis

The samples were divided into high- and low-risk groups based on the median risk score, and the differences in OS and progression-free survival between the high-risk and low-risk groups were estimated using the Kaplan–Meier method. Survival curves were compared using the log-rank test. The significance threshold was defined as *p* < 0.05.

### Statistical Analysis

Statistical analyses were performed using the log-rank test for univariate analysis. Pearson’s correlation test was used to assess the relationship between the risk score and immune markers, characteristic gene expression, and the immune cell infiltration score. The relationship between the characteristic gene expression and the immune cell infiltration score was also evaluated. Student’s *t*-tests were used to determine statistical significance of differences between variables. Statistical significance was defined as *p* < 0.05. All statistical analyses were performed in R version 4.0.2.

## Results

### Identification of the Differentially Expressed Homeobox Gene Family Members in Bladder Cancer

**FIGURE 2:**
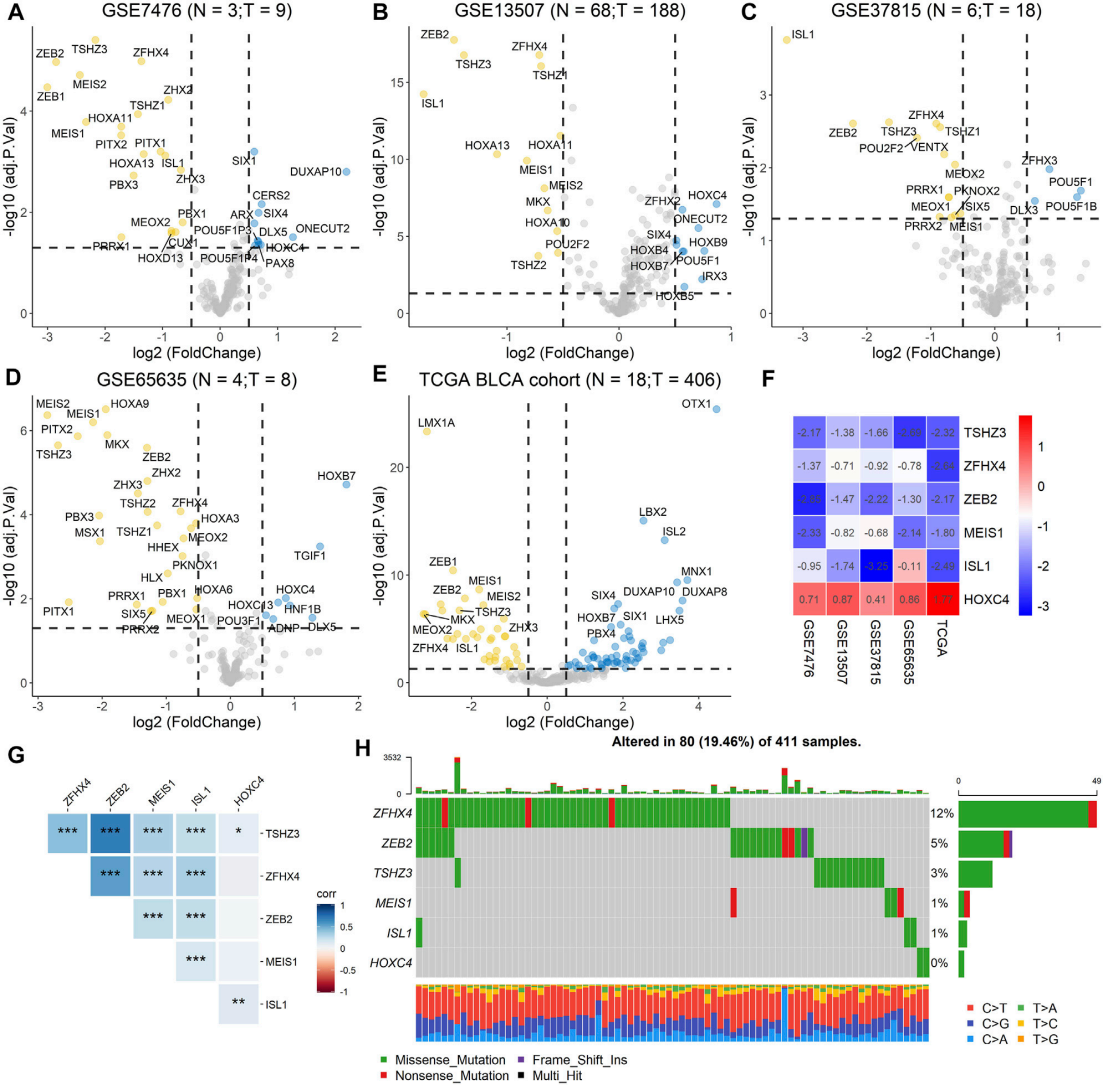
Identification of DEHGs for BLCA, analysis of the mutation landscape, and correlation analysis of the six DEHGs. **(A)** DEHGs for BLCA in the GSE7476 dataset. **(B)** DEHGs for BLCA in the GSE13507 dataset. **(C)** DEHGs for BLCA in the GSE37815 dataset. **(D)** DEHGs for BLCA in the GSE65635 dataset. **(E)** DEHGs for BLCA in the TCGA dataset. **(F)** LogFC values of each gene in different datasets (GSE7476, GSE13507, GSE37815, GSE65635, and TCGA). **(G)** Analysis of the correlations among the six DEHGs. **(H)** Mutation landscape of the six DEHGs in TCGA BLCA patients.

To screen differentially expressed homeobox genes (DEHGs) in BLCA, four GEO datasets, GSE7476, GSE13507, GSE37815, and GSE65635, as well as TCGA gene expression dataset containing 406 BLCA samples and 18 normal samples from the UCSC Xena Browser were obtained. The R package “limma” was used to determine the DEHGs of each dataset using |logFC| > 0.5 and adjusted *p* < 0.05 criteria, and the volcanoes were plotted (Figures 2A–E). Furthermore, the RRA method based on the expression of each gene in all datasets was used to screen out the candidate genes (score < 0.05) (Supplementary Table 1). As a result, six homeobox genes, TSHZ3, ZFHX4, ZEB2, MEIS1, ISL1, and HOXC4, were screened out, and then the logFC values of each gene in different datasets were calculated and are shown in Figure 2F. Moreover, correlation analysis of the six homeobox genes was performed, and the results showed that there were significant positive correlations between most genes (Figure 2G).

### Correlation of the Six Homeobox Genes With Clinical Status and Mutation Landscape

**FIGURE 3:**
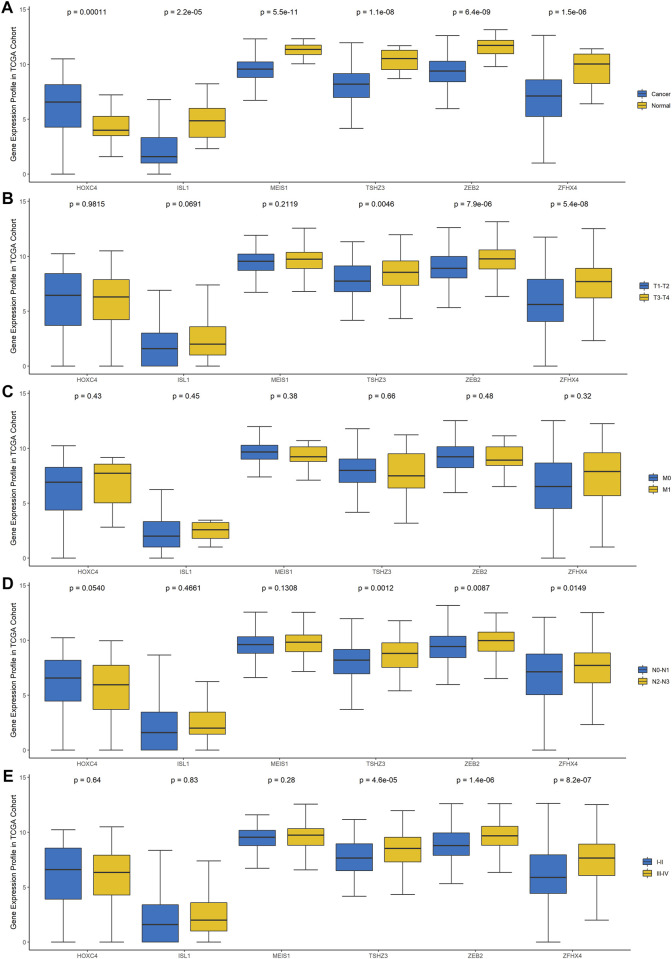
Gene expression profile of these six genes in TCGA cohort. **(A)** Differences in the expression of the six genes between BLCA tissues and normal tissues. **(B)** Differences in the expression of the six genes in different T stages. **(C)** Differences in the expression of the six genes in different N stages. **(D)** Differences in the expression of the six genes in different M stages. **(E)** Differences in the expression of the six genes in different clinical stages.

To explore the clinical significance of these six genes, pancancer analysis in BLCA (Figure 3A) and 23 other tumors (Supplementary Figure 1) was performed, and the results revealed that the expression of TSHZ3, ZFHX4, ZEB2, MEIS1, and ISL1 was significantly lower than that in normal tissues, while the expression of HOXC4 was higher than that in normal tissues, especially in BLCA, breast invasive carcinoma (BRCA), prostate adenocarcinoma (PRAD), and head and neck squamous cell carcinoma (HNSC). Furthermore, we analyzed the correlation between these six homeobox genes and tumor size, regional lymph node involvement, and distant metastases (TNM) as well as the BLCA stage and found that TSHZ3, ZFHX4, and ZEB2 were positively correlated with T stage, N stage, and BLCA stage, but there was no significant correlation with metastasis (Figures 3B–E). In addition, we analyzed the mutation landscape of these six DEHGs in BLCA. Among the 411 samples, 19.46% had at least one gene mutation; ZFHX4 mutation was the most common change, accounting for 12% of mutations; ZEB2, TSHZ3, MEIS1, and ISL1 mutations accounted for 5, 3, 1, and 1% of all mutations, respectively. The waterfall diagram formed according to the mutation landscape of these six DEHGs showed that most mutations were missense mutations (Figure 2H). The driver genes ERBB2, HDAC1, PARP1, ERBB3, FGFR3, mTOR, AXL, EZH2, FGFR1, FGFR2, CSF1R, KIT, FGFR4, RET, and ERBB4 are key targets in the treatment of BLCA. Furthermore, we assessed the correlations between these six genes and BLCA driver genes in the BLCA dataset, and it was found that these six genes have a strong correlation with these driver genes (Supplementary Figure 2).

### A High Risk Score Was Associated With a Poor Clinical Outcome

**FIGURE 4:**
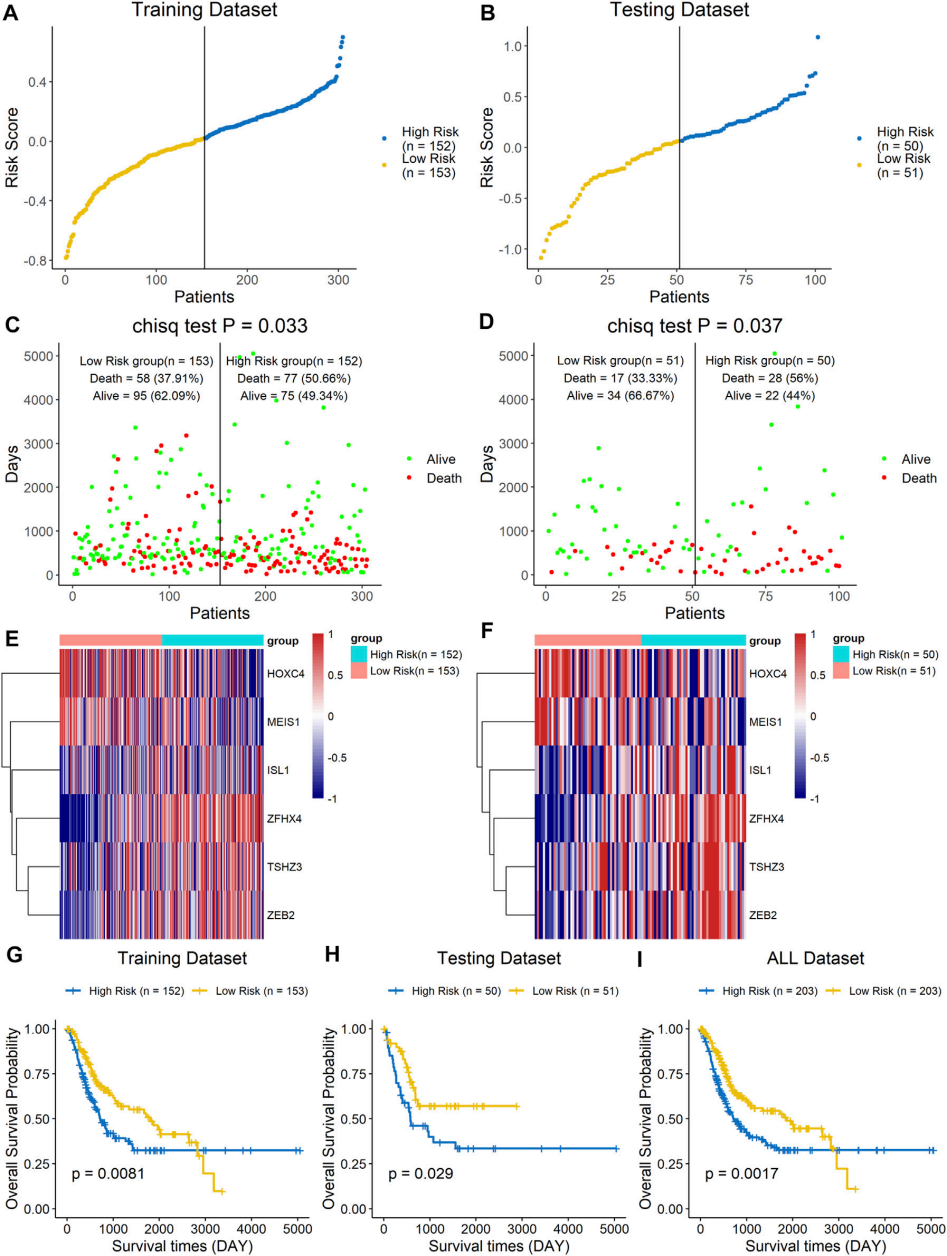
A high risk score was associated with a poor clinical outcome. The BLCA cohort was divided into two groups based on the median estimated score, and the two groups were then compared. The ranked dot plot indicates the risk score distribution in the training dataset **(A)** and testing dataset **(B)**. Scatter plot presenting the patients’ overall survival status in the training dataset **(C)** and testing dataset **(D)**. Heat map with the gene expression profiles of these six genes in the training dataset **(E)** and testing dataset **(F)**. Kaplan–Meier curve analysis of the signature in the training set **(G)**, testing set **(H)**, and entire dataset **(I)**.

The prognostic value of the six-homeobox-gene signature was evaluated in the training dataset and testing dataset. We calculated the risk score for each BLCA sample in the training set, ranked them according to this score, and divided them into high-risk and low-risk groups based on the median risk score. We used scatter plots to show the survival status of BLCA patients based on risk scores, and we then performed a chi-square test on the data (Figures 4A,C). The results demonstrated that patients in the high-risk group had a higher mortality rate than those in the low-risk group (*p* = 0.033). The heat map with the gene expression profile of these six homeobox genes showed that ISL1, ZFHX4, TSHZ3, and ZEB2 were more highly expressed in high-risk BLCA samples, while HOXC4 and MEIS1 were highly expressed in the low-risk group (Figure 4E). The results for the testing dataset were consistent with those for the training dataset (Figures 4B,D,F). Kaplan–Meier analysis was performed on the training dataset, the testing dataset, and all datasets (Figures 4G–I), and the results revealed that the survival time of the low-risk group was significantly longer than that of the high-risk group.

### GO Function Annotation and KEGG Pathway Analysis Between the High-Risk and Low-Risk Groups

**FIGURE 5:**
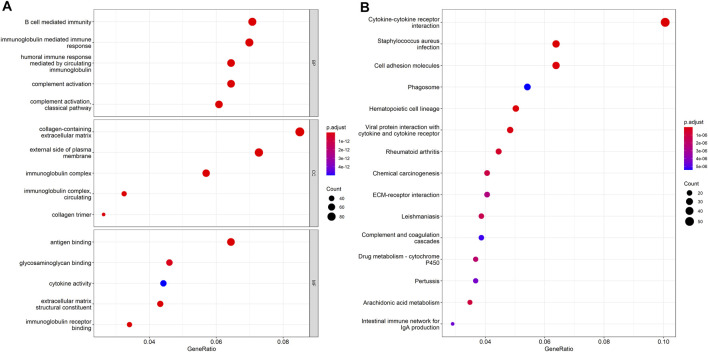
Functional enrichment between the high-risk and low-risk groups. **(A)** GO function annotation. **(B)** KEGG pathway analysis.

The DEHGs between the two risk groups were analyzed using GO functional annotation and KEGG pathway analysis with the R software package “clusterProfiler.” The GO analysis of biological process (BP), molecular function (MF), and cell component (CC) terms showed that most of the enriched terms were related to immunity, including B cell-mediated immunity, immunoglobulin-mediated immune response, immunoglobulin complex, and antigen binding (Figure 5A). The KEGG pathway analysis showed that the DEHGs were mainly enriched in cytokine-cytokine receptor interactions, *Staphylococcus aureus* infection, cell adhesion molecules, etc., most of which are related to immunity (Figure 5B).

### The Signature Composed of Six Homeobox Genes Was Closely Related to Immunity

**FIGURE 6:**
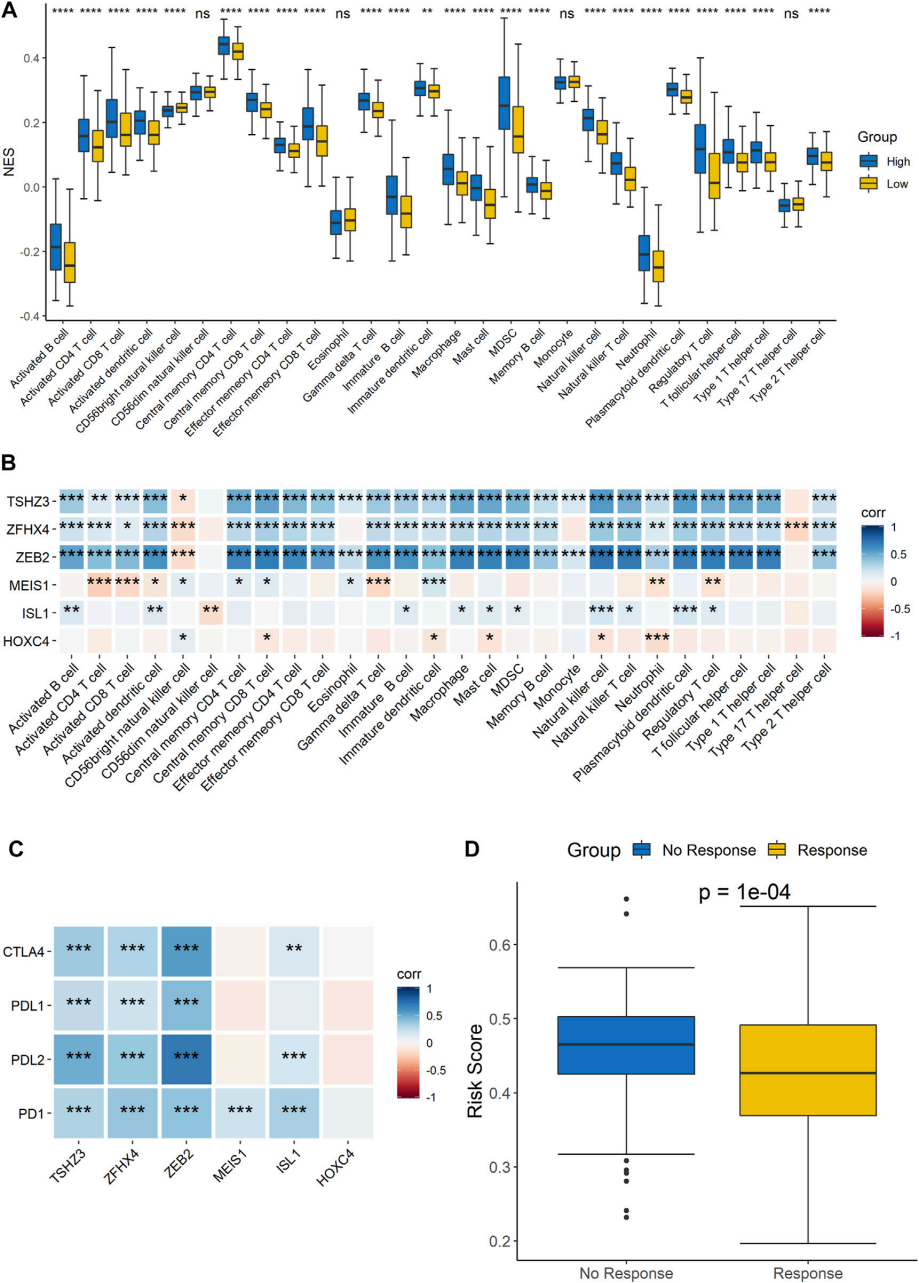
Correlation analysis of the signature and immune characteristics. **(A)** Correlations between the signature and each immune cell infiltration score. **(B)** Correlations between each signature gene and each immune cell infiltration score. **(C)** Correlations between the expression level of immune checkpoints and the six signature genes. **(D)** Prediction of the difference in risk scores between immunotherapy responders and non-responders.

Since the results of the GO functional annotation and KEGG pathway analysis showed that the signature was related to immunity, analysis of the risk score and immune cell infiltration was then performed to further confirm the conclusion. The results showed that there were differences in the infiltration of most immune cells, except for CD56dim natural killer cells, eosinophils, and monocytes, between the high- and low-risk groups, which demonstrated that the signature was significantly correlated with immune infiltration (Figure 6A). In addition, we analyzed the correlation of each gene with the infiltration of immune cells, and the results indicated that TSHZ3, ZFHX4, and ZEB2 were related to almost all immune cell types and that MEIS1, ISL1, and HOXC4 were related to some immune cell types (Figure 6B). Furthermore, we also analyzed the correlation analysis between these six genes and cytokines related to T cell function. The results showed that five out of the six genes, TSHZ3, ZFHX4, ZEB2, MEIS1, and ISL1, had a strong correlation with most cytokines, while HOXC4 had a strong correlation with IL-17A (Supplementary Figure 3). Similarly, the analysis of the correlations between the expression of these six homeobox genes and immune checkpoints showed that TSHZ3, ZFHX4, and ZEB2 were significantly correlated with the expression of CTLA-4, PD-L1, PD-L2, and PD-1. MEIS1 was strongly correlated with the expression of PD-1. In addition, ISL1 was significantly correlated with CTLA-4, PD-L2, and PD-1 expressions (Figure 6C). Then, we analyzed the relationship between the risk score and the response to immunotherapy. The samples were divided into response and no-response groups, and the difference in risk scores between the two groups was assessed. The results showed that the risk scores were higher in the no-response group than in the response group (Figure 6D).

### Evaluation and External Validation of the Signature Model Performance

**FIGURE 7:**
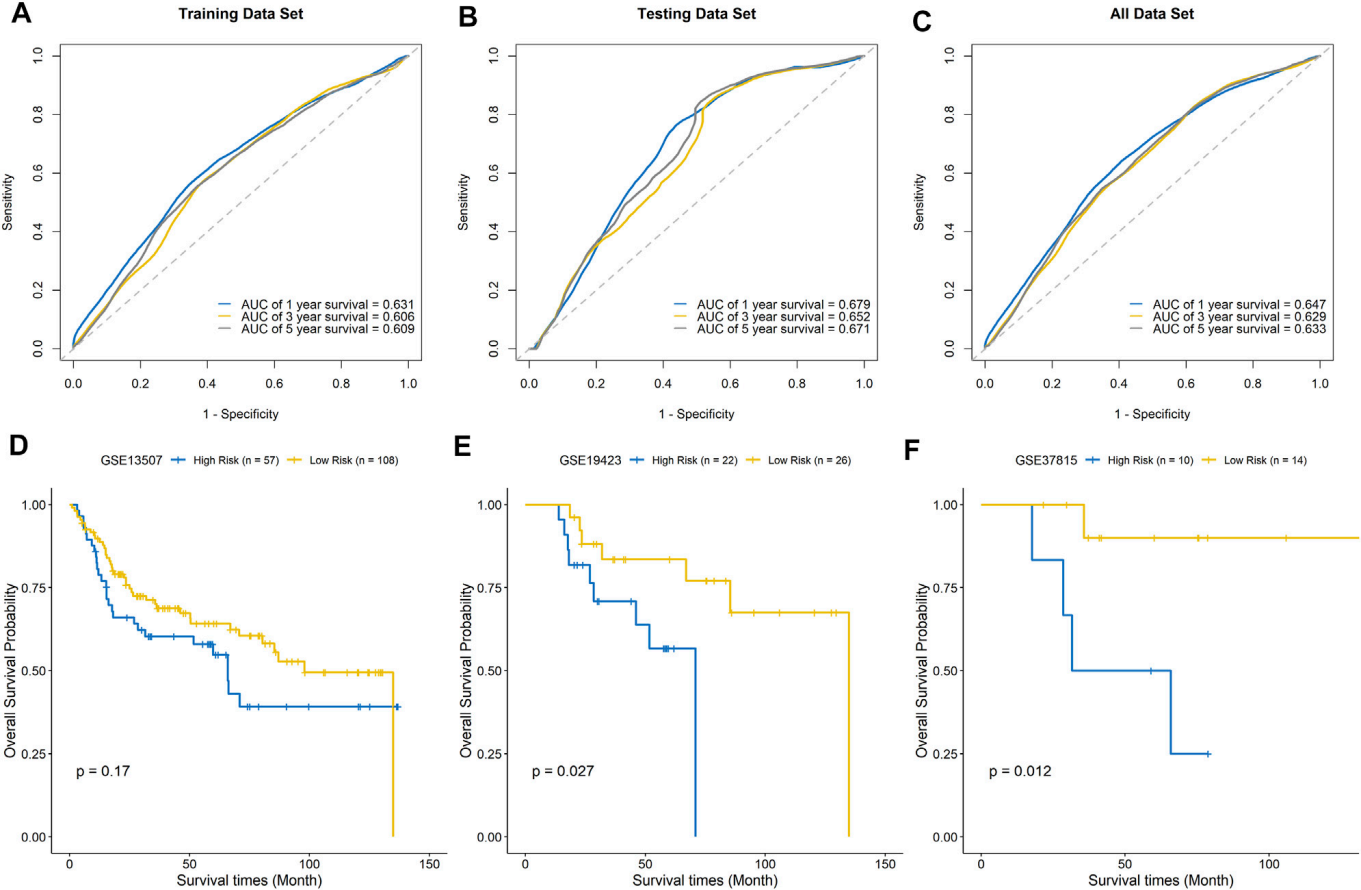
Evaluation of the signature model. ROC curves for predicting one-, three-, and five-year survival in the training set **(A)**, testing set **(B)**, and entire dataset **(C)**. External validation of the signature model using the GEO BLCA cohorts GSE13507 **(D)**, GSE19423 **(E)**, and GSE37815 **(F)**.

The ROC curves of the training set, testing set, and entire dataset (combination of training and testing sets) were plotted, and the area under the ROC curve (AUC) was calculated to verify the accuracy of this signature. The AUCs for one-, three-, and five-year OS were 0.631, 0.606, and 0.609 in the training set; 0.679, 0.652, and 0.671 in the testing set; and 0.647, 0.629, and 0.633, respectively, in the entire dataset (Figures 7A–C). To compare the consistency of the model predictions with actual clinical outcomes, calibration curves for one-, three-, and five-year OS were constructed for the training set (Supplementary Figures 4A–C), testing set (Supplementary Figures 4D–F), and entire dataset (Supplementary Figures 4G–I). The calibration curves showed satisfactory agreement between the predicted and observed values for one-, three-, and five-year OS. We further validated the prediction ability of this prognostic signature using the GEO datasets GSE13507, GSE19423, and GSE37815 for external validation. The risk score of each sample was calculated, and the samples were divided into high-risk and low-risk groups based on the optimal splitting point. Kaplan–Meier analysis of GSE13507 (*p* = 0.17), GSE19423 (*p* = 0.027), and GSE37815 (*p* = 0.012) showed that the high-risk group tended to have a shorter survival time than the low-risk group (Figures 7D–F).

## Discussion

There are many studies on biomarkers of BLCA, such as urine cytology and urine biomarkers; the detection of exfoliated tumor cells in urine or bladder lavage has a high sensitivity for the diagnosis of high-grade BLCA but is less sensitive for low-grade BLCA. There are many biomarkers with unique functions, such as radiotherapy markers, chemotherapy markers, and immunotherapy markers, but these markers have a single function (Giordano and Soria, 2020), and most of them involved single targets, which easily cause false-positive or false-negative results. The application of RNA-Seq and bioinformatic analysis of databases has provided a theoretical basis for mechanistic studies of tumorigenesis and development. Zhu et al. identified some immune-related genes as prognostic factors in BLCA (Zhu et al., 2020). Lian et al. established a signature including eight long non-coding RNAs as a candidate prognostic biomarker for BLCA (Lian et al., 2019). At present, there are few biomarkers that can predict both clinical outcomes and immunotherapy response. In this study, a clinical prediction model containing six homeobox genes was constructed through next-generation sequencing (NGS), which can not only predict the prognosis of patients but also predict the patient’s immune response. With the popularity of sequencing technology, its price and convenience continue to improve, and this study has good clinical applicability. Although the homeobox gene family is closely related to BLCA (Cantile et al., 2011), few studies have focused on its prognostic value in BLCA. Therefore, we analyzed the RNA-Seq data of a large number of samples from TCGA and GEO public databases and screened out six significant DEHGs, namely, TSHZ3, ZFHX4, ZEB2, MEIS1, ISL1, and HOXC4, by the RRA method.

Some of these six homeobox genes have been reported to regulate tumor progression and were identified as potential prognostic markers in previous studies. For example, aberrant HOXC4 expression is prevalent and plays an important role in the development of prostate cancer (Luo and Farnham, 2020). Moreover, HOXC4 can promote hepatocellular carcinoma progression by transactivating Snail (Yang et al., 2021). The expression of TSHZ3 is significantly downregulated in human glioma tissues and cell lines, and overexpression of TSHZ3 decreases the invasiveness of U87 and U251 glioblastoma cells (Li et al., 2018). In addition, the downregulation or deletion of TSHZ3 function is involved in the pathogenesis of ovarian cancer (McBride et al., 2012), which suggests that TSHZ3 plays an oncogenic role. ZFHX4 is required for the regulation of glioblastoma tumor–initiating cells, and its inhibition leads to reduced tumorigenesis and increased glioma-free survival time. Mutations in ZFHX4 are strongly associated with a poor prognosis, and downregulation of ZFHX4 inhibits the progression of esophageal squamous carcinoma (Qing et al., 2017). ZEB2 can promote the migration and invasion of gastric cancer cells by regulating epithelial–mesenchymal transition (EMT) and is a potential target for gene therapy of invasive gastric cancer (Dai et al., 2012). Deregulation of negative feedback between GATA3 and ZEB2 can promote breast cancer metastasis (Si et al., 2015). The expression level of MEIS1 in acute myeloid leukemia (AML) is negatively correlated with prognosis (Rozovskaia et al., 2001). ISL1 plays an important role in a variety of cellular processes, including cytoskeleton genesis, organogenesis, and tumorigenesis (Zheng and Zhao, 2007), and has been found to be a highly specific marker for pancreatic endocrine tumors and metastases (Schmitt et al., 2008). In addition, it was also significantly associated with aggressive tumor characteristics, tumor recurrence, tumor progression, and disease-specific mortality (DSM) in BLCA and plays an important role in multiple stages of bladder tumorigenesis (Akhir et al., 2020).

We constructed a predictive signature based on these six prognostic homeobox genes. The expression profiles of the signature genes showed that tumors with higher risk scores tended to exhibit elevated ISL1, ZFHX4, TSHZ3, and ZEB2 levels, while those with lower risk scores tended to exhibit elevated HOXC4 and MEIS1 levels. Patients with high risk scores according to the signature had a poor prognosis. Then, we performed survival analysis on the training dataset, the testing dataset, and all datasets. The results showed that the high-risk group had a shorter survival time than the low-risk group. Finally, we validated the performance of the signature using GEO datasets. Overall, the signature can predict the prognosis of patients accurately and has good prognostic value.

Errors in the process of DNA replication are random and universal and subject to correction and repair by the DNA mismatch repair system. Once the dynamic balance between the two is disrupted, it will easily lead to the occurrence of gene mutations, which will affect the expression of the corresponding genes and facilitate tumorigenesis and development (Turajlic et al., 2019). We analyzed the mutation landscape of these six genes in BLCA. Among the 411 samples, 19.46% had at least one gene mutation. Driver genes are important genes associated with tumor development and play a driving role in the process of cancer development and progression (Martínez-Jiménez et al., 2020). Currently, the driver genes of BLCA include ERBB2, HDAC1, PARP1, and mTOR. These genes are important targets in BLCA treatment (Scholtes et al., 2020). We performed correlation analysis between these six genes and BLCA driver genes in the BLCA dataset and found that these six genes have a strong correlation with the driver genes. The results indicated that the six homeobox genes play an important role in the development of BLCA and that the signature could be used in the prediction of BLCA prognosis.

As a major component of the tumor microenvironment (TME), immune infiltration has been shown to contribute to tumor progression and the immunotherapeutic response (Balkwill et al., 2012), and tumor-infiltrating immune cells, particularly T cells, are the cellular basis of immunotherapy. A better understanding of immune cells in the TME is critical to deciphering the mechanisms of immunotherapy, defining predictive biomarkers, and identifying new therapeutic targets (Zhang and Zhang, 2020; Ma et al., 2021). In our GO analysis, most of the enriched functional terms were immune-related, and the same results were obtained by KEGG analysis. Then, the analysis of the risk score and immune cell infiltration showed that there were differences in the infiltration of most immune cells between the high- and low-risk groups, which demonstrated that this signature was significantly correlated with immune infiltration. Immune cells in tumors work together to control tumor growth, and the effectiveness of immunotherapy depends on the synergistic response of innate and adaptive immune cells, particularly T cells (Moynihan et al., 2016). The function of T cells is usually classified based on whether they secrete specific effector molecules or cytokines, and effector CD4^+^ T cells include different functional subtypes (Th1 cells secrete IL-2 and IFN-γ; Th2 secretes IL-4, -13; Th17 secretes IL-17A, etc.), while effector CD8^+^ T cells secrete cytotoxic mediators (perforin and granzymes) or proinflammatory cytokines (TNF-α, IFN-γ) (Szabo et al., 2019). Therefore, in order to analyze the correlation between these six genes and T cell function, we further analyzed the correlation of these six genes with those cytokines, and the results showed that five out of the six genes, TSHZ3, ZFHX4, ZEB2, MEIS1, and ISL1, had a strong correlation with most cytokines, while HOXC4 had a strong correlation with IL-17A. The expression of the six homeobox genes in the signature was correlated with most immune checkpoints (CTLA-4, PD-L1, PD-L2, and PD-1). At present, the most commonly used immunotherapy drugs in clinical practice are immune checkpoint inhibitors. Therefore, we analyzed the relationship between the risk score and the response to immunotherapy. The results showed that the risk scores were higher in the no-response group than in the response group. Above all, this signature was highly correlated with immunity and will be a good predictor of the patient’s response to immunotherapy.

However, this study has some limitations. This study is based on TCGA and GEO databases, the reliability of its data is unknown, and this study lacks experimental evidence and is mostly based on bioinformatics prediction, which limits its immediate applicability in clinical practice. In addition, the number of non-tumor tissues assessed in this study is rather small (*n* = 18), which constitutes a potentially important bias influencing the results. The GEO datasets used for validation were relatively small. Further validation of model prediction accuracy with clinical data is needed.

## Conclusion

We constructed a risk prediction signature with six homeobox genes, which showed good accuracy and consistency in predicting the patient’s prognosis and the response to immunotherapy. Therefore, this signature could be a potential biomarker and treatment target for BLCA patients.

## Data Availability

The datasets presented in this study can be found in online repositories. The names of the repository/repositories and accession number(s) can be found in the article/Supplementary Material.

## Supplementary Material

Supplementary Figure 1 Pancancer analysis of the six homeobox genes.

Supplementary Figure 2 Correlation analysis of the six homeobox genes and BLCA driver genes.

Supplementary Figure 3 Correlation analysis of these six genes and cytokines related to T cell function.

Supplementary Figure 4 Calibration curves of the signature model in predicting one-, three-, and five-year OS in the training set **(A–C)**, testing set **(D–F)**, and entire dataset **(G–I)**.

Supplementary Table 1 Results based on the RRA method.
