# Supplementary figures and images for: Identification of a Prognostic Signature Associated With the Homeobox Gene Family for Bladder Cancer
Source: Front Mol Biosci. 2021 Jul 21;8:688298. doi: 10.3389/fmolb.2021.688298 (PMC8334560; doi:10.3389/fmolb.2021.688298)

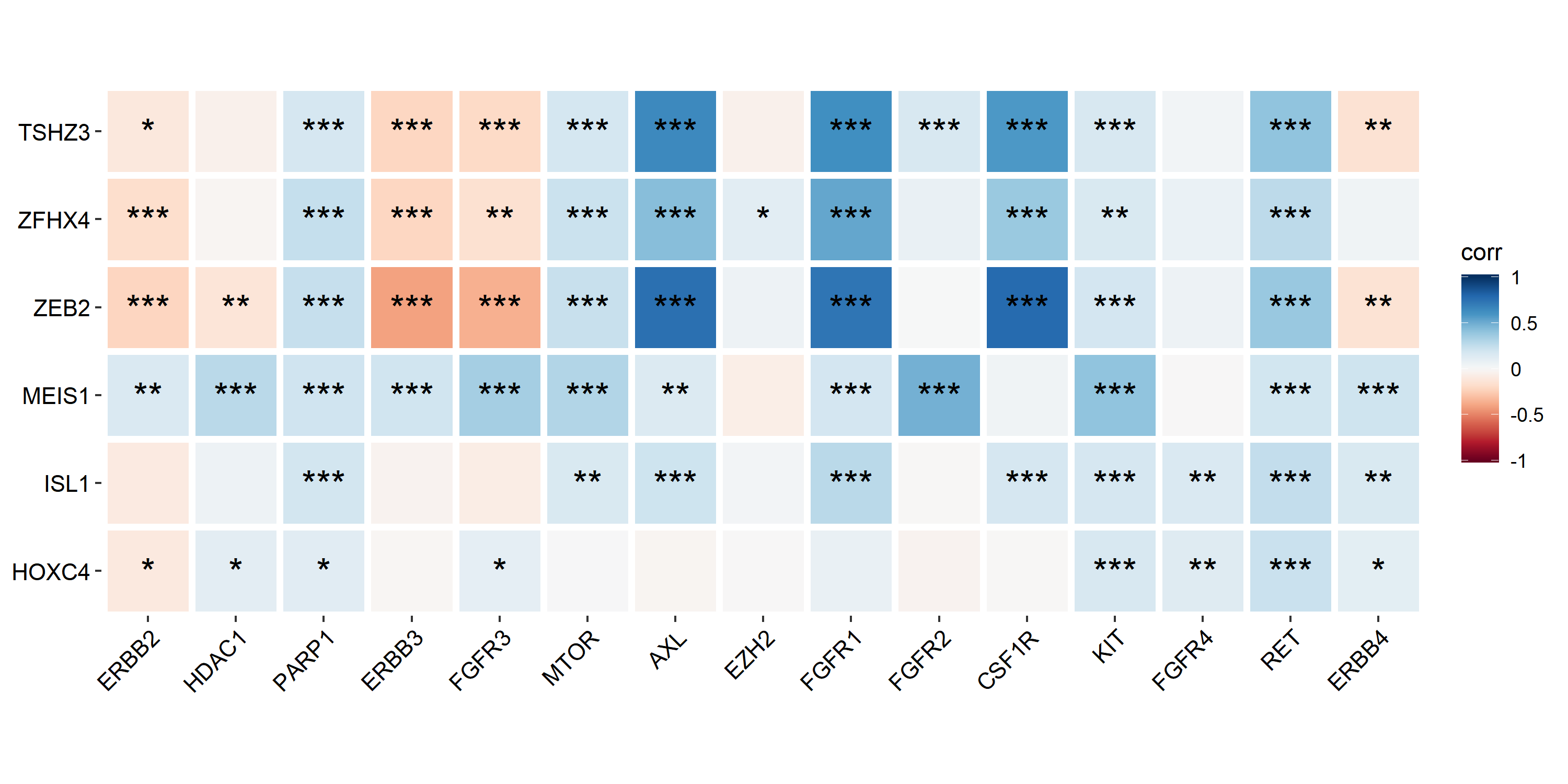

Supplement: Supplementary file 2 [file Image2.TIFF]

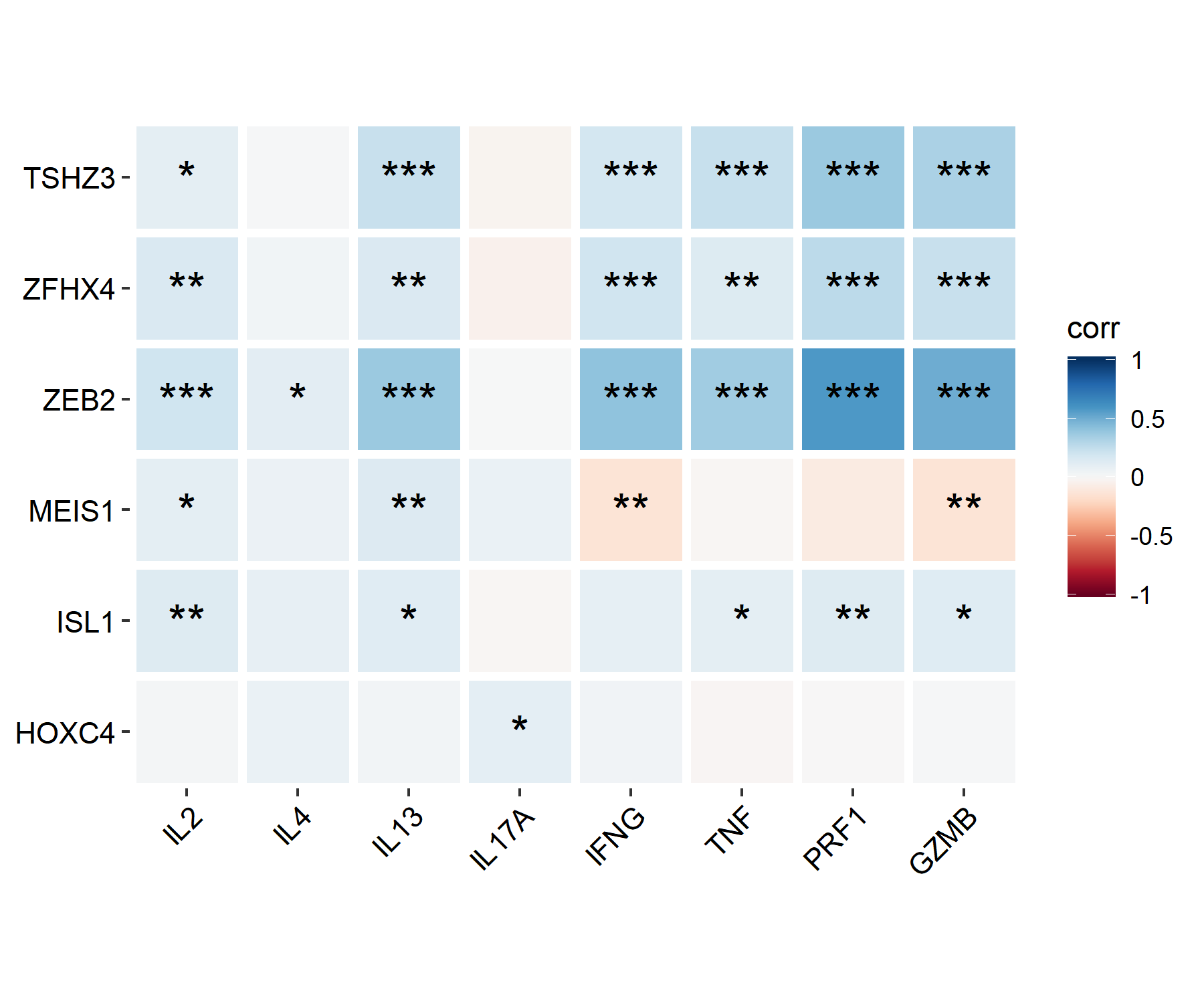

Supplement: Supplementary file 3 [file Image3.TIFF]

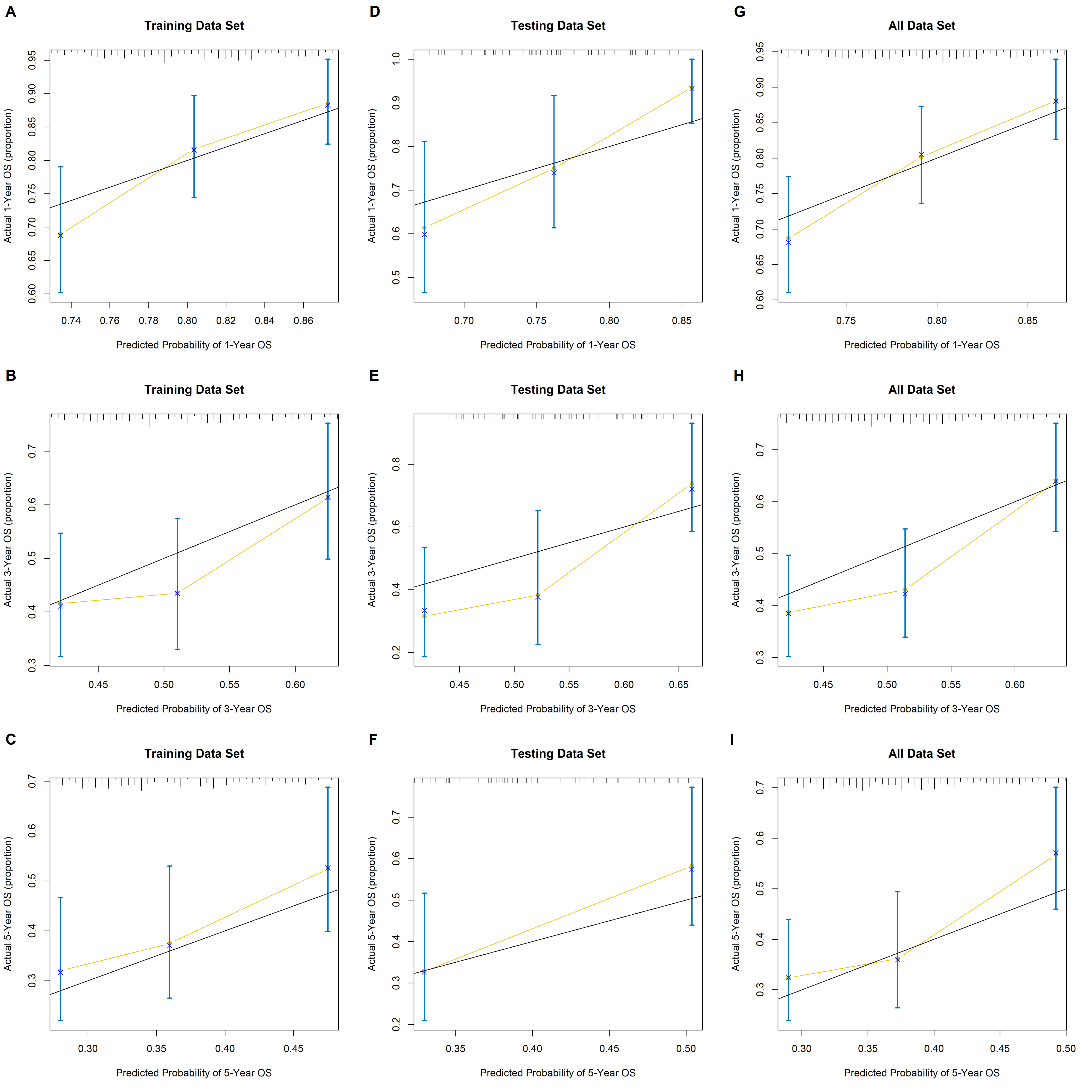

Supplement: Supplementary file 4 [file Image4.TIFF]
